# Warming delays photosynthesis downregulation but not growth in a boreal conifer

**DOI:** 10.1093/plphys/kiaf325

**Published:** 2025-07-23

**Authors:** Laura Fernández-de-Uña

**Affiliations:** Assistant Features Editor, Plant Physiology, American Society of Plant Biologists; Department of Plant Biology and Soil Sciences, Universidade de Vigo, 32004 Ourense, Spain

Plants in higher latitudes adjust their activity (e.g. photosynthesis and tissue development) in response to intra-annual changes in environmental conditions, such as the number of light hours (i.e. photoperiod), radiation intensity, temperature, or water availability. This intra-annual variability leads to physiological activity that is highly reduced in winter, when temperatures, photoperiod, and incoming radiation are low, and peaks around the summer solstice, when radiation levels are optimal and temperatures and water availability have not yet become limiting. While these phenological adjustments in plant function are obvious in deciduous species, evergreen species also modulate their physiological activity in response to intra-annual environmental variability.

Woody plants increase in height (primary growth) and in stem and branch diameter (secondary growth) annually. Stem growth entails the production of new xylem cells, which in conifers consist mainly of tracheids that have both water-transport and structural-support functions. Temperature directly affects cambial cell division, enlargement, and differentiation processes. Low temperatures inhibit xylem growth by reducing the concentration and sensitivity of growth hormones such as auxins, cytokinins, and gibberellins, inducing cytoskeleton microtubule depolymerization, and decreasing cell-wall lignification (Rathgeber et al. 2022, and references therein). Water availability can also affect xylem formation processes through its effect on cell turgor (Abe et al. 2003). The number and features (e.g. diameter, cell-wall width) of xylem cells affect its hydraulic and mechanical properties, including its water-transport efficiency and resistance to drought- or frost-induced embolism (i.e. gas-filled conduits that disrupt water flow) (Hacke and Sperry 2001).

Low temperatures also inhibit the carboxylation reactions of photosynthesis. However, energy absorption continues, causing photo-oxidative damage unless photoprotective mechanisms are activated (Huner et al. 1998). Carotenoids such as xanthophyll cycle pigments not only complement chlorophyll during photosynthesis by absorbing additional light wavelengths, but they also dissipate excess light as heat through a mechanism called non-photochemical quenching (Huner et al. 1998). As the growing season comes to an end, evergreen leaves alter their chlorophyll and carotenoid concentrations to better sustain winter conditions, downregulating photosynthesis and upregulating photoprotection (Huner et al. 1998).

Phenological studies in both deciduous and evergreen species have largely focused on the environmental factors driving plant activity resumption after the winter (e.g. Menzel et al. 2006; Delpierre et al. 2019). However, our understanding of the environmental factors signaling the end of the growing season is more limited and at times contradictory, partly due to the greater interactions occurring among factors and the more gradual processes involved, such as cell-wall thickening or leaf cold acclimation. Daylength and minimum temperatures have been suggested to signal the arrival of unfavorable conditions. Drought conditions during the summer may also halt primary and secondary growth due to limiting turgor and carbon availability and abscisic acid signaling (Huang et al. 2021; Rathgeber et al. 2022). This cessation may be temporary, with growth resuming once water availability increases (Vieira et al. 2015), or it may mark the end of xylem growth (Fernández-de-Uña et al. 2017) and even the growing season (Wu et al. 2022). The observed and projected increase in global temperatures and related increase in vapor pressure deficit and drought severity are already altering, and expected to further change, the seasonal cycles in plant activity. Warming in temperate and boreal ecosystems has generally resulted in the lengthening of the growing season (Menzel et al. 2006). Yet, species that maintain a photoperiod control over their leaf or cambial phenology or plants experiencing increasingly drier conditions may not be able to profit from temperature-induced growing season extension.

Recently in *Plant Physiology*, Murphy et al. moved forward in our understanding of how autumn phenology may change under climate change scenarios by analyzing how experimental mid-summer drought and autumn warming affected photosynthesis downregulation, growth phenology, and xylem properties in white spruce (*Picea glauca*) seedlings from two different provenances (northern and southern Eastern Canada). Warming delayed the downregulation of photosynthesis and the upregulation of photoprotection in both families (Murphy et al. 2025). On the other hand, moderate summer drought did not affect the timing of photosynthetic downregulation. This suggests low temperatures are the main cue for needle cold acclimation. However, photoprotective upregulation occurred in the warming treatment under higher temperatures than under control conditions, suggesting a significant photoperiod control. Thus, temperatures may be the primary factor signaling needle winter acclimation, but if the activating threshold is not reached, photoperiod acts as a secondary trigger to avoid cold and photo-oxidative damage.

Conversely, the phenology of shoot and secondary growth was unaffected by the warming and drought treatments (Murphy et al. 2025). The lack of a drought effect on stem growth contrasts with studies that have found xylem growth is more sensitive to drought than leaf processes (Fernández-de-Uña et al. 2017; Huang et al. 2021) and may indicate that the moderate drought applied, which mimics climate change projections in the area, may not limit growth in white spruce. These results also suggest that photoperiod exerts significant control over xylem formation in this species and that either the applied warming was not able to overcome its signal or that growth had already ceased by the time warming was applied and it was not enough to reactivate meristem division.

Xylem anatomical properties responded to the treatments differently in the two families assessed. Cell wall reinforcement and relative wood density, both related to the proportion of cell wall versus lumen, were lower in control seedlings of the southern family than in the northern family (Murphy et al. 2025). Both variables increased in the earlywood as a result of summer drought and in the latewood as a result of warming in the southern family but decreased in the northern one. The southern family also delayed photosynthesis downregulation more than the northern one and may have invested part of the excess carbon in xylem safety rather than storage.

Murphy et al. show that the phenology of photosynthesis was more sensitive to autumn warming than the phenology of primary and secondary growth. While current year growth was unaffected by the delay in photosynthetic downregulation, future research should explore how the delay in photosynthetic downregulation affects carbon reserves and how stored carbon is allocated, either during the current growing season or the following one.

## Data Availability

No new data were generated or analysed in support of this article.
